# Exploration of serum biomarkers in heart failure patients with preserved and reduced ejection fractions through analysis of heterogeneity^☆^

**DOI:** 10.1016/j.bbrep.2025.102183

**Published:** 2025-07-31

**Authors:** Qipeng Jin, Zhicheng Wang, Wenyong Lin, Chunling Zhang, Xiaolong Wang

**Affiliations:** Branch of the National Clinical Research Center for Chinese Medicine Cardiology, Shuguang Hospital Affiliated with Shanghai University of Traditional Chinese Medicine, China

**Keywords:** Heart failure, Serum metabolomics, WGCNA, Biomarker

## Abstract

**Background:**

Heart failure with preserved ejection fraction (HFpEF) and heart failure with reduced ejection fraction (HFrEF) differ markedly in their pathophysiology. Distinguishing HFpEF remains challenging, particularly among patients with hypertension and metabolic syndrome.

**Methods:**

Untargeted metabolomics using liquid chromatography-tandem mass spectrometry (LC-MS/MS) was performed on serum samples from four groups: healthy controls (N), hypertensive patients with diabetes (H_A_T), HFpEF, and HFrEF. Key metabolites distinguishing HFpEF were identified using pathway enrichment analysis and random forest machine learning. ROC curve analysis evaluated their diagnostic accuracy.

**Results:**

A total of 3924 metabolites were identified. Amino acid and fatty acid metabolic pathways emerged as central to heart failure pathophysiology. Metabolites including DG(13:0/20:3(8Z,11Z,14Z)/0:0), glutarylcarnitine, homo-l-arginine, sphingosine, and (R)-3-hydroxybutyrylcarnitine were highlighted as diagnostic markers. A predictive model based on these metabolites effectively distinguished HFpEF from other subtypes, with enhanced specificity when combined with NT-proBNP.

**Conclusions:**

The identified serum metabolites show promise as early diagnostic biomarkers for HFpEF. Validation in larger, multicenter cohorts and further investigation into underlying biological mechanisms are necessary to confirm their clinical utility.

## Highlights

• Metabolomics identifies distinct biomarkers for HFpEF diagnosis.
• Novel metabolites enhance diagnostic specificity for HFpEF.
• Metabolite-based models improve early differentiation of HFpEF from HFrEF.
• Combining metabolites with NT-proBNP optimizes HFpEF diagnosis accuracy.

## Graphical abstract

**Figure:**
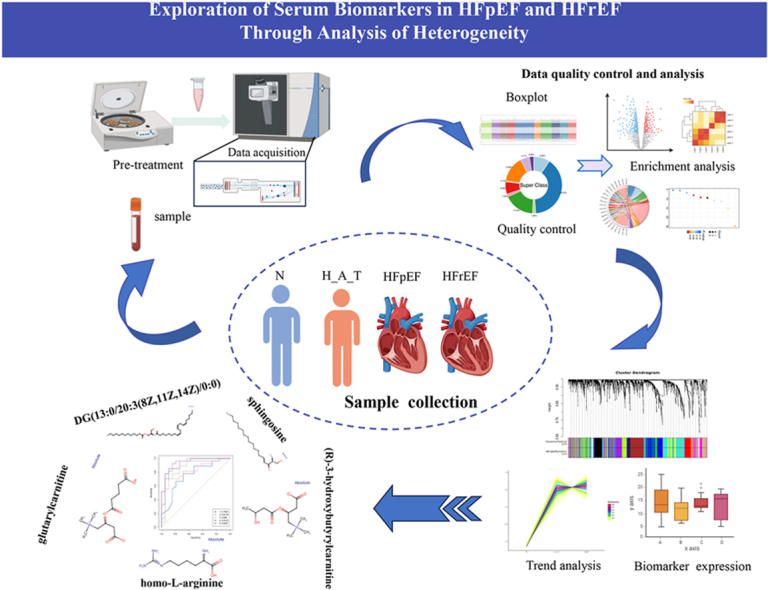

## 1 Introduction

Heart failure (HF) represents a global health challenge, with significant morbidity and mortality affecting approximately 64 million individuals worldwide. The increasing incidence of HF, driven by aging populations and increasing cardiovascular risk factors, necessitates a better understanding of its subtypes. HF can be classified on the basis of the left ventricular ejection fraction (LVEF) into heart failure with a reduced ejection fraction (HFrEF, LVEF <40 %), heart failure with a preserved ejection fraction (HFpEF, LVEF ≥50 %), and heart failure with a mid-range ejection fraction (HFmrEF, LVEF 40–49 %) [1]. More than 50 % of HF patients have HFpEF, and this proportion continues to increase. However, the pathophysiology of HFpEF remains poorly understood, particularly its relationship with metabolic syndrome. This study seeks to address this gap by applying untargeted metabolomics combined with machine learning algorithms to systematically explore HFpEF-specific biomarkers, providing new insights for early diagnosis and individualized treatment.

The incidence of HFpEF is closely related to premature mortality [2]. Initially (approximately 1975–1995), HFpEF was considered a sequela of hypertensive heart disease. However, changes in modern lifestyles have led to a two-to threefold increase in the prevalence of diabetes in the general population, along with a significant increase in proinflammatory comorbidities, increasing the diversity of the clinical features of HFpEF, which is often accompanied by comorbidities such as diabetes, hypertension, chronic kidney disease, and coronary artery disease. Hypertension is a major risk factor for HFpEF [3], with a prevalence of 55 %–90 % among HFpEF patients. Central and peripheral mechanisms induced by hypertension, such as left ventricular hypertrophy, diastolic dysfunction, fibrosis, left atrial enlargement, and large vessel and microvascular stiffening, are believed to be important etiologies of HFpEF. Diabetes is another key risk factor for HFpEF. In the CHARM study [4], 40 % of HFpEF patients were diagnosed with diabetes at enrollment, with an additional 22 % in a prediabetic state with HbA1c levels between 6.0 % and 6.4 %. Epidemiological studies have also shown that one-third of HFpEF patients are diagnosed with diabetes. Oxidative stress, vascular inflammation, and endothelial dysfunction caused by multiple factors play central roles in the pathophysiological development of HFpEF, which is increasingly recognized as a type of heart failure closely associated with metabolic syndrome.

Therefore, in this study, we systematically selected four key groups of controls, patients with hypertension and diabetes, HFpEF patients, and HFrEF patients explore the differences and heterogeneity in their serum biomarkers. These groups represent different stages, from a healthy state to a significant pathological condition. Although the metabolic pathophysiology of HFpEF has been preliminarily studied, a significant research gap remains in the application of multidimensional analysis via metabolomics combined with machine learning algorithms, especially in the context of HFpEF heterogeneity and its clinical translation. This study is the first to systematically apply untargeted metabolomics combined with random forest algorithms to explore specific biomarkers and their role in HFpEF. We selected the normal control group as a baseline to assess metabolite changes in pathological states. By analyzing the metabolic profiles of healthy individuals, we aim to identify biomarkers that change significantly during disease progression, potentially playing a key role in the development of heart failure. The inclusion of the hypertension and diabetes group is designed to elucidate the early metabolic effects of major heart failure risk factors before clinical symptoms arise. Given that hypertension and diabetes are core risk factors for HFpEF, an in-depth analysis of this population's metabolic characteristics may reveal early contributions to the pathophysiological process of HFpEF and provide clues for identifying early biomarkers. HFpEF patients are the central focus of this study because of their complex pathological mechanisms and diverse comorbidity profiles, making the heterogeneity of this group particularly prominent.

Using untargeted metabolomics, we comprehensively analyzed the serum metabolite profiles of HFpEF and HFrEF patients, aiming to identify HFpEF-specific biomarkers and explore their potential pathological mechanisms. The inclusion of HFrEF patients allows for a direct comparison of the metabolic characteristics of the two heart failure subtypes, revealing their essential differences in pathophysiology. This study aimed to systematically compare the metabolic profiles of the four groups to identify key metabolic pathways and markers related to the heterogeneity of HFpEF and HFrEF. These findings not only deepen our understanding of the complex pathophysiology of HFpEF but also potentially lay the foundation for the development of individualized therapeutic strategies in the future.

## 2 Methods

### 2.1 Study design

Between August 2023 and June 2024, we recruited participants from Shuguang Hospital, Affiliated with Shanghai University of Traditional Chinese Medicine and collected clinical and demographic data along with plasma samples. We enrolled 31 patients with hypertension and diabetes who were diagnosed according to the 2018 European Society of Hypertension/European Society of Cardiology (ESH/ESC) guidelines for hypertension and the World Health Organization (WHO) and American Diabetes Association (ADA) criteria for diabetes management [5,6]. Hypertension was defined as clinic blood pressure ≥140/90mmHg or ambulatory blood pressure monitoring daytime average ≥ 135/85 mmHg, with further classification into Grade 1 (140–159/90–99 mmHg), Grade 2 (160–179/100–109 mmHg), and Grade 3 (≥180/110 mmHg). Diabetes was diagnosed on the basis of fasting blood glucose ≥7.0 mmol/L, HbA1c ≥ 6.5 %, 2-h blood glucose ≥11.1 mmol/L during the oral glucose tolerance test, or random blood glucose ≥11.1 mmol/L with diabetic symptoms.

In addition, we recruited 117 chronic heart failure patients, including 89 HFpEF patients and 28 HFrEF patients. The diagnosis followed the 2021 European Society of Cardiology guidelines [7] and was based on typical symptoms (dyspnea, fatigue, and peripheral edema), signs (jugular venous distension, pulmonary rales, and lower extremity edema), and laboratory and imaging examinations. Heart failure was classified as HFrEF (LVEF <40 %), HFmrEF (LVEF 40–49 %), or HFpEF (LVEF ≥50 %) on the basis of LVEF. The laboratory tests included natriuretic peptide (NT-proBNP >125 pg/mL), along with echocardiographic assessment of LVEF, ventricular size, myocardial thickness, and valve function. The sample size was determined via published scientific guidelines. The detailed inclusion and exclusion criteria for all study groups and the diagnostic process for HFpEF are provided in the supplementary materials (see Supplementary Information).

### 2.2 Sample collection and preparation

Fasting blood samples were collected in the early morning following an overnight fast of at least 10 h, in EDTA anticoagulant tubes, to minimize the influence of recent dietary intake and circadian variation. Samples were immediately placed on ice and centrifuged at 3000 rpm for 15 min at 4 °C. The plasma was aliquoted into 1.5 mL EP tubes, with 500 μL of plasma per tube, to reduce freeze-thaw cycles, quickly snap-frozen in liquid nitrogen, and stored in a −80 °C freezer until analysis.

Prior to metabolomic extraction, all samples were thawed simultaneously in an ice‒water mixture to ensure uniform handling. Then, 100 μL of each sample was transferred into a 1.5 mL EP tube, followed by the addition of 400 μL of methanol (Fisher, A4524) and acetonitrile (Fisher, A998-4) for protein precipitation (2:1, v/v; containing internal standards at 4 μg/mL). The samples were vortexed for 1 min and subjected to ultrasonic extraction in an ice–water bath for 10 min. After standing at −40 °C for 2 h, the samples were centrifuged at 13,000 rpm for 20 min at 4 °C. Subsequently, 150 μL of the supernatant was filtered through a 0.22 μm organic phase needle filter and transferred to an LC injection vial for further analysis.All procedures were performed under cold conditions to minimize metabolic degradation and variability. UPLC‒MS analysis was performed via a Waters ACQUITY UPLC I-Class plus/Thermo QE HF system for ultrahigh-performance liquid chromatography-tandem mass spectrometry (LC-MS/MS).

### 2.3 Data analysis

Metabolomic data analysis began with quality control (QC) via internal standards and QC samples, followed by principal component analysis (PCA), hierarchical clustering analysis, correlation analysis, and evaluation of metabolite intensity distributions. The raw data were preprocessed via Progenesis QI v3.0 software (Nonlinear Dynamics, Newcastle, UK), including baseline filtering, peak identification, integration, retention time correction, peak alignment, and normalization. Compound identification was based on multiple dimensions, including retention time, accurate mass, MS/MS fragmentation, and isotopic distribution, via the HMDB, Lipid maps (v2.3), and METLIN databases. Ion peaks with an RSD greater than 0.3 were removed. For missing or zero values, ion peaks with more than 50 % missing data within a group were deleted, and the remaining zero values were imputed with half of the lowest ion intensity in the dataset. After log2 transformation, compounds were filtered on the basis of score, and those with a score lower than 36 (out of 80) were excluded. The identified compounds were classified into four levels, with priority given to Level 1 and Level 2 compounds for further validation.

#### 2.3.1 Basic statistical analysis

Clinical and demographic data were analyzed via IBM SPSS Statistics 25.0. Continuous variables were tested for normality and homogeneity of variance. Normally distributed data are expressed as the means ± standard deviations, whereas nonnormally distributed data are presented as interquartile ranges. Group comparisons were performed via one-way ANOVA, and nonnormally distributed data were tested via the Shapiro‒Wilk test. Paired t-tests were used for comparisons within normally distributed groups, and the Wilcoxon signed-rank test was used for nonnormally distributed data. Independent sample t tests were used for pre-and posttreatment comparisons, with statistical significance set at P < 0.05.

#### 2.3.2 Multivariate statistical analysis

Multivariate statistical analysis of metabolomics data included unsupervised PCA to observe sample distribution and assess process stability, followed by orthogonal partial least squares discriminant analysis (OPLS-DA) to analyze differences in metabolic profiles between groups. To minimize the risk of overfitting, 7-fold cross-validation and 200-times permutation tests were performed to validate the OPLS-DA models. Differentially abundant metabolites were initially identified based on p < 0.05, fold change (FC) ≥1.2 or ≤0.83, and variable importance in projection (VIP) scores. For downstream Kyoto Encyclopedia of Genes and Genomes (KEGG) pathway enrichment analyses, significant metabolites were further filtered using FDR-adjusted p-values (q < 0.05) to control for multiple testing. Sensitivity analyses excluding potential outliers identified by Cook's distance were also performed to assess the robustness of the results. No missing data were present after preprocessing.

#### 2.3.3 WGCNA and trend analysis

Weighted gene coexpression network analysis (WGCNA) [8] and trend analysis were employed to identify coexpression patterns of metabolites and their dynamic changes in four groups (normal, hypertension with diabetes as an intermediate state, HFrEF, and HFpEF). The soft-thresholding power (β) was selected based on achieving a scale-free topology fit index (signed R^2^) exceeding 0.85. WGCNA was first used to standardize the metabolite data and construct a weighted network. The topological overlap matrix (TOM) was used to assess metabolite relationships and identify metabolite modules. Module eigengenes (MEs) were calculated to correlate modules with phenotypes such as sex, age, and LVEF, and key metabolite modules were identified. Trend analysis by clustering algorithms was performed to identify dynamic changes in metabolites across different disease states, and statistically significant metabolites were further analyzed through Kyoto Encyclopedia of Genes and Genomes (KEGG) enrichment, revealing the heterogeneity between HFpEF and HFrEF.

#### 2.3.4 Biomarker selection and model construction

We applied a random forest model combined with cross-validation to identify the most robust biomarkers capable of distinguishing between HFpEF and HFrEF. By analyzing the importance of the features, we selected the top five metabolites and further evaluated their diagnostic performance via receiver operating characteristic (ROC) curve analysis.

**Fig. 1:**
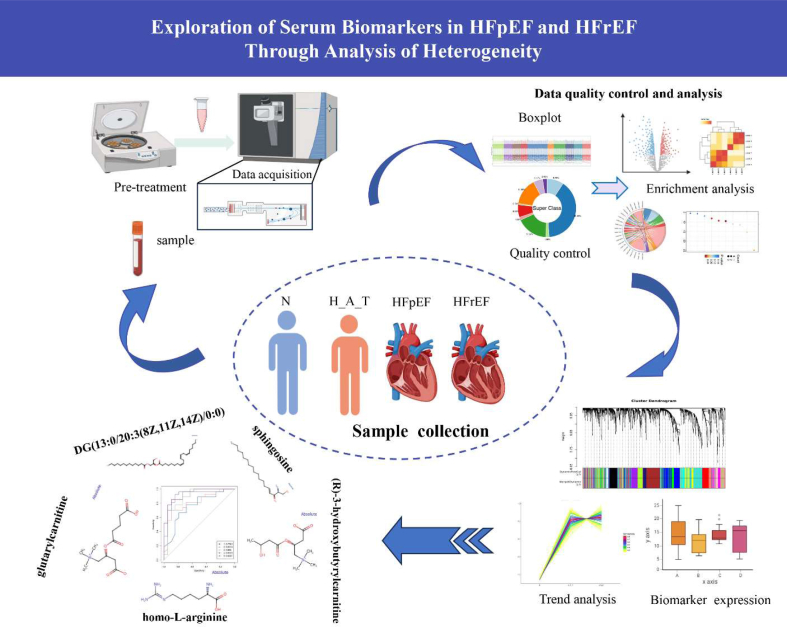
Workflow of biomarker analysis in HFpEF and HFrEF (Graphical Abstract).

Fig. 1 summarizes the study process: sample collection, LC-MS/MS for data acquisition, data analysis, and biomarker discovery.

## 3 Results

### 3.1 Comparison of baseline clinical characteristics

A total of 30 healthy controls (N), 31 patients with hypertension and diabetes (H-A-T), 89 patients with HFpEF, and 28 patients with HFrEF were included in this study, with all participants providing informed consent. There was no statistically significant difference in sex among the four groups. In terms of age, the mean ages of the HFpEF group (70.12 ± 6 years) and the HFrEF group (71.96 ± 5.34 years) were significantly greater than those of the H-A-T group (62.42 ± 6.76 years, p < 0.01) and the N group (60.3 ± 7.6 years, p < 0.01), but there was no significant difference between the HFpEF and HFrEF groups (Table 1).

**Table 1:** Demographic characteristics and clinical indicators of the participants.

Variable	Normal(n = 30)	Hypertension & diabetes(n = 31)	Heart failure with preserved ejection fraction (n = 89)	Heart failure with reduced ejection fraction (n = 28)	*P* value				
					Overall	Normal-H&D	H&D-HFpEF	H&D-HFrEF	HFpEF–HF–rEF
Age,y	60.3 ± 7.6	62.42 ± 6.76	70.12 ± 6	71.96 ± 5.34	<0.01	0.19	<0.01	<0.01	0.18
Sex									
Female,n(%)	14(46.67)	11(35.48)	38(42.70)	12(42.8)	0.84	0.26	0.43	0.37	0.58
Male,n(%)	16(53.33)	20(64.51)	51(57.3)	16(57.2)					
Medications									
ACEi/ARB, n (%)	0(0)	31(100)	68(76.40)	28(100)	<0.01	<0.01	<0.01	<0.01	<0.01
CCB, n(%)	0(0)	5(16.13)	33(37.07)	1(3.57)	<0.01	<0.01	<0.01	<0.01	<0.01
β-Blocker, n (%)	0(0)	5(16.13)	40(44.90)	24(85.71)	<0.01	<0.01	<0.01	<0.01	<0.01
Loop diuretic, n (%)	0(0)	1(3.2)	18(20.22)	23(82.14)	<0.01	<0.01	<0.01	<0.01	<0.01
New Oral Anticoagulants, n(%)	0(0)	0(0)	32(35.96)	13(46.43)	<0.01	NA	<0.01	<0.01	0.21
Statin,n(%)	0(0)	22(64.51)	40(44.94)	17(60.71)	<0.01	<0.01	<0.01	0.29	<0.01
Echocardiography									
Left ventricular ejection fraction, %	65.5(64,66.25)	64(63,67)	62(60,65)	38(36,39)	<0.01	0.99	<0.01	<0.01	<0.01
E/e’ ratio	6.66 ± 1.21	7.18 ± 1.31	11.08 ± 1.97	15.78 ± 2.38	<0.01	0.27	<0.01	<0.01	<0.01
left atrial volume index	31.04 ± 3.16	31.59 ± 3.33	41.62 ± 3.28	39.12 ± 4.72	<0.01	0.54	<0.01	<0.01	<0.01
aboratory data									
NT-proBNP, pg/mL	89.13 ± 30.20	94.04 ± 29.62	933.84 ± 334.27	4359.23 ± 1892.31	<0.01	0.98	<0.01	<0.01	<0.01
growth stimulating express gene 2, ng/ml	18.39 ± 4.46	22.20 ± 3.28	22.83 ± 7.01	38.12 ± 8.60	<0.01	0.01	0.64	<0.01	<0.01
Hemoglobin A1C, %	5.42 ± 0.28	6.97 ± 1.28	6.23 ± 1.10	6.8 ± 1.51	<0.01	<0.01	<0.01	0.56	0.02
Serum creatinine, mg/dL	64.69 ± 12.37	70.89 ± 13.93	72.48 ± 23.61	83.59 ± 24.22	<0.01	0.25	0.71	0.02	0.01
low-density lipoprotein, mg/dl	2.32 ± 0.56	2.39 ± 0.68	2.18 ± 0.96	2.06 ± 0.93	0.41	0.75	0.23	0.14	0.52

Laboratory indicators revealed that the NT-proBNP levels in chronic heart failure patients were significantly greater than those in patients in the N- and H-A-T groups and the NT-proBNP levels in the HFrEF group were greater than those in the HFpEF group, which is consistent with the clinical manifestations of HFrEF patients. Additionally, the soluble growth stimulation factor levels in the HFrEF group were greater than those reported in previous studies; although these levels were slightly elevated in the H-A-T group, they remained within the normal range. Compared with the other two groups, the H-A-T and HFrEF groups presented significantly elevated HbA1c levels.

In the assessment of kidney function, the serum creatinine levels in the HFrEF group were significantly greater than those in the other three groups, reflecting poorer metabolic function. The echocardiographic results indicated that the LVEF in the HFrEF group (36.93 ± 2.65 %) was significantly lower than that in the other three groups. Although there were significant differences in LVEF among the other three groups, their LVEF remained within the normal range. The E/e' ratio increased with increasing severity of heart failure, with significant differences among the four groups. The LAVI values were greater in the HFpEF group, reflecting the pathological characteristics of HFpEF, whereas in the HFrEF group, the LAVI decreased as the LVEF decreased. These clinical differences provide a reliable basis for the diagnosis and treatment of different types of heart failure.

### 3.2 Quality control (QC) in LC-MS/MS metabolomics

**Fig. 2:**
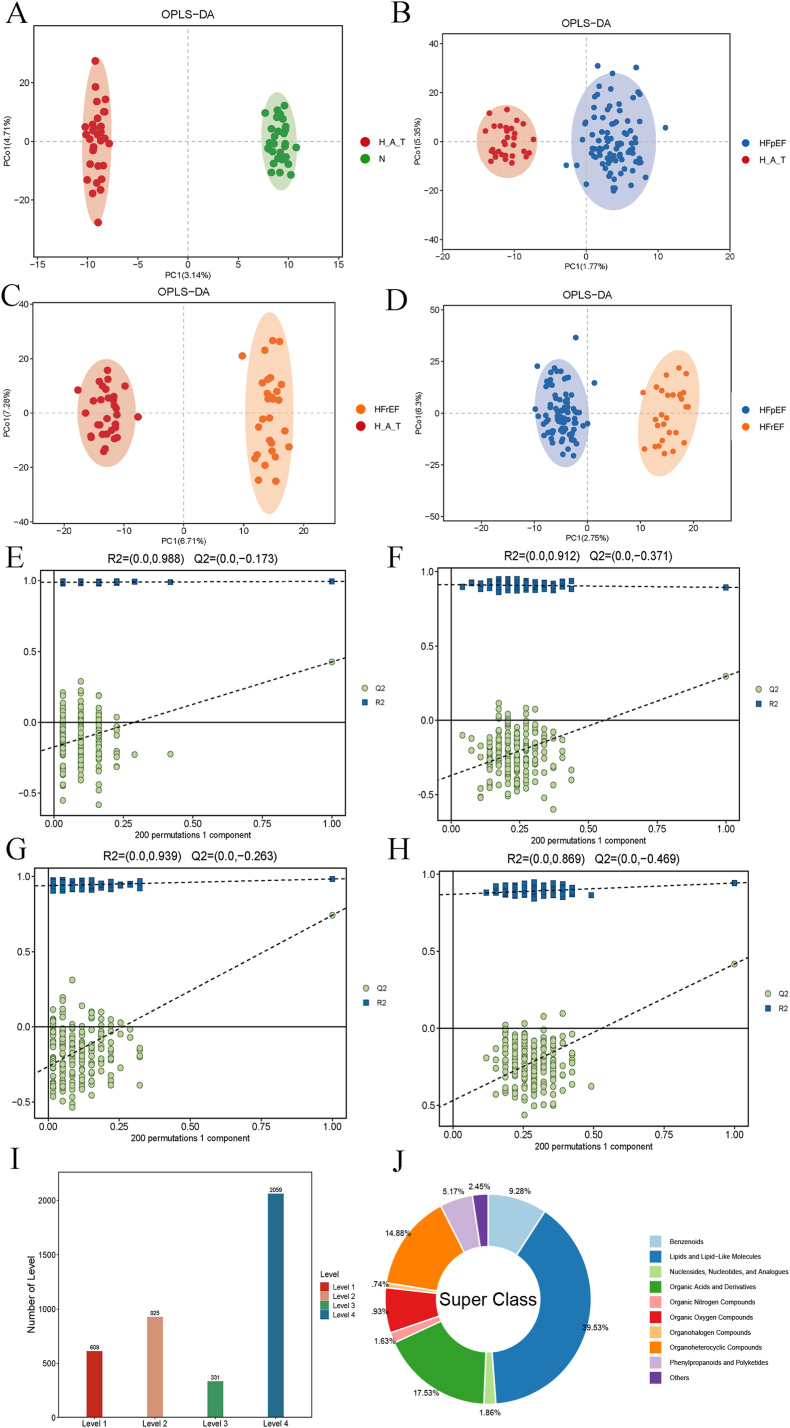
OPLS-DA score plots and permutation test results of metabolic profiles among different groups and classification of identified metabolites. A–D: OPLS-DA score plots comparing the metabolic profiles of A: H_A_T vs. N, B: H_A_T vs. HFpEF, C: H_A_T vs. HFrEF, and D: HFpEF vs. HFrEF. Each ellipse represents the 95 % confidence interval for each group, showing separation between the different groups. E–H: Permutation test results for the OPLS-DA models corresponding to E: H_A_T vs. N, F: H_A_T vs. HFpEF, G: H_A_T vs. HFrEF, and H: HFpEF vs. HFrEF, demonstrating model robustness (R^2^) and predictive power (Q^2^). I: Distribution of identified metabolites across different confidence levels (Levels 1–4). J: Superclass classification of the identified metabolites, showing the proportions of various metabolite classes.

After seven cycles of verification, the PCA results revealed that the QC samples were tightly clustered, indicating the stability of mass spectrometry detection and the reproducibility of the experiment. To further explore the relationship between metabolite expression levels and sample grouping, we applied OPLS-DA to construct a model. The OPLS-DA score plots demonstrated clear separation among the four groups, highlighting significant intergroup metabolic differences. To rigorously assess model validity and minimize the risk of overfitting, we performed 200-times permutation tests. The results showed that all permuted Q^2^ values were substantially lower than those of the original models and distributed below zero (Supplementary Fig. S2), indicating that the OPLS-DA models were stable and not overfitted. This comprehensive evaluation provides confidence that the observed group discrimination reflects genuine metabolic differences rather than modeling artifacts. ((Fig. 2). A-D & E-H). Overall, LC-MS/MS analysis identified a total of 3924 metabolites spanning 19 major metabolite categories. (Fig. 2I and J).

### 3.3 Differences in metabolite classifications among plasma samples from different groups

**Fig. 3:**
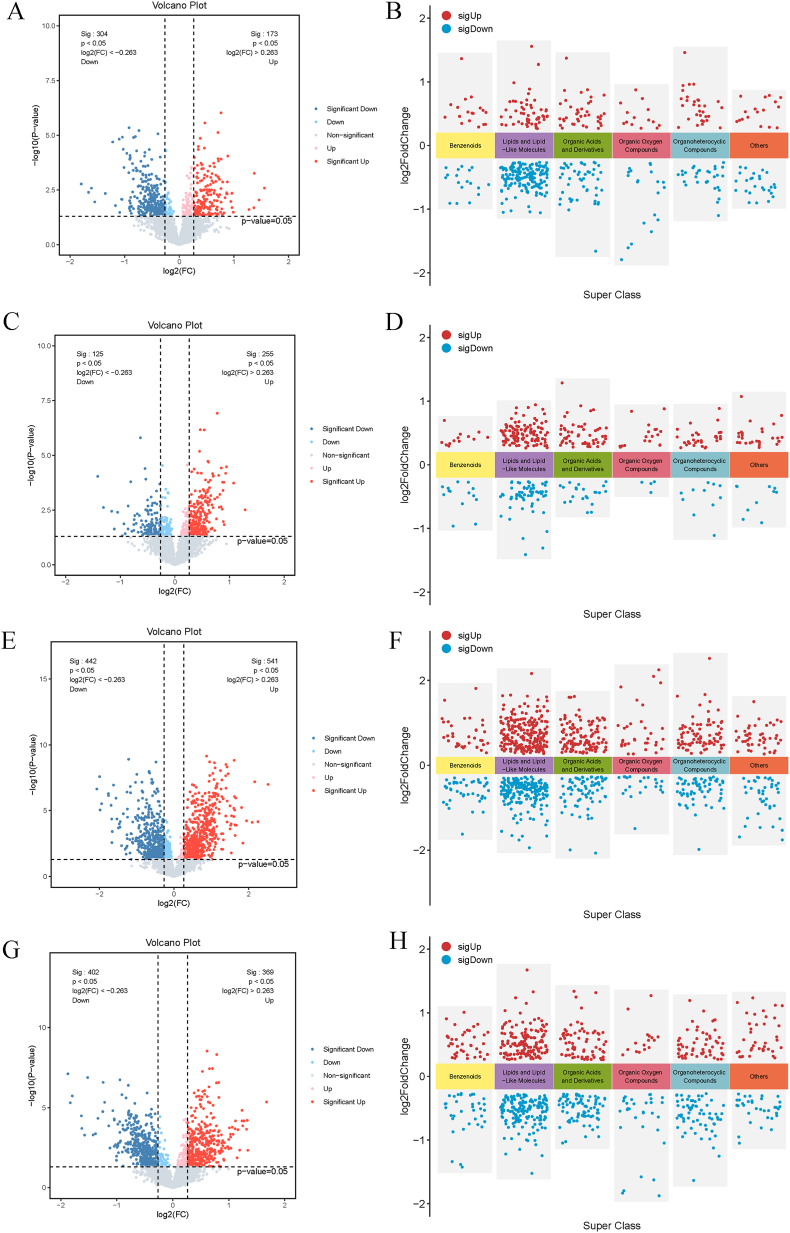
Volcano plots and superclass distributions of significantly altered metabolites across comparisons. A–D: Volcano plots showing the log2-fold change (log2FC) vs. -log10 p-value for each metabolite. Red dots represent significantly upregulated metabolites, and blue dots represent significantly downregulated metabolites (p < 0.05). A: H_A_T vs. N, B: HFpEF vs. H_A_T, C: HFrEF vs. H_A_T, D: HFpEF vs. HFrEF. E–H: Superclass distribution of significantly altered metabolites, color-coded by upregulation (red) or downregulation (blue). Metabolites are grouped into superclasses, including benzenoids, lipids, organic acids, and others. E: H_A_T vs. N, F: HFpEF vs. H_A_T, G: HFrEF vs. H_A_T, H: HFpEF vs. HFrEF.

The analysis revealed significant metabolic differences between the groups. Comparing the H_A_T group with the N group, 477 differentially abundant metabolites were identified, mainly involving lipids and lipid-like molecules, as well as organic acids and their derivatives, with a significant downregulation of lipid metabolism. In the comparison between the HFpEF group and the H_A_T group, 380 differentially abundant metabolites were detected, revealing an increase in lipid and organic acid metabolites, suggesting a shift in the metabolic environment. When the HFrEF group was compared with the H_A_T group, as many as 983 differentially abundant metabolites were identified, with notable changes in both the upregulation and downregulation of lipid metabolism, indicating more complex metabolic regulation in HFrEF. A comparison between the HFrEF and HFpEF groups revealed 771 differentially abundant metabolites, with significant differences in lipid and organic acid metabolism (Fig. 3).

### 3.4 KEGG pathway analysis of differential metabolites

**Fig. 4:**
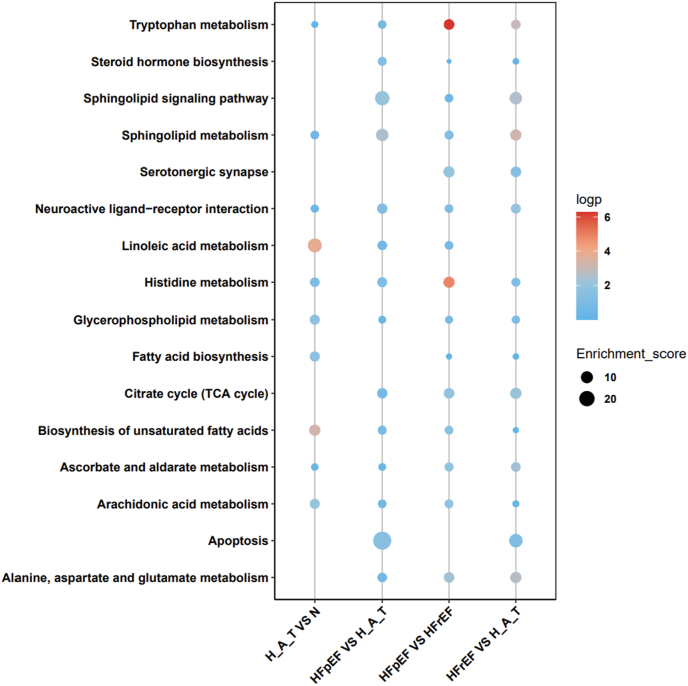
KEGG pathway enrichment analysis across groups.

We conducted pairwise comparisons across four groups, using hypertension combined with diabetes as an intermediate state of metabolic disorder, to explore the progressive transition from a normal metabolic state to metabolic dysfunction and, eventually, heart failure. The results revealed significant alterations in several key metabolic pathways as the disease progressed. Notably, enrichment in amino acid metabolism and fatty acid metabolism was particularly pronounced, highlighting the central role of metabolic dysregulation in the pathophysiology of heart failure. In further analyses, we compared the metabolic and signaling pathway differences between two distinct types of heart failure (HFpEF and HFrEF). The results revealed that HFpEF is closely associated with lipid metabolism disorders and energy imbalance, especially in pathways related to sphingolipid metabolism and fatty acid biosynthesis, suggesting that disruptions in lipid and energy metabolism are central to the pathogenesis of HFpEF. In contrast, HFrEF was more strongly linked to severe inflammatory responses and cellular dysfunction following metabolic dysregulation, with notable abnormalities in glutamate metabolism and fatty acid oxidation pathways, which further exacerbated myocardial injury and dysfunction in HFrEF patients (Fig. 4).

The dot plot shows KEGG pathway enrichment for four group comparisons: H_A_T vs. N, HFpEF vs. H_A_T, HFpEF vs. HFrEF, and HFrEF vs. H_A_T. Dot colors reflect the -log(p value) (blue to red), while sizes represent enrichment scores.

### 3.5 WGCNA and trend analysis

**Fig. 5:**
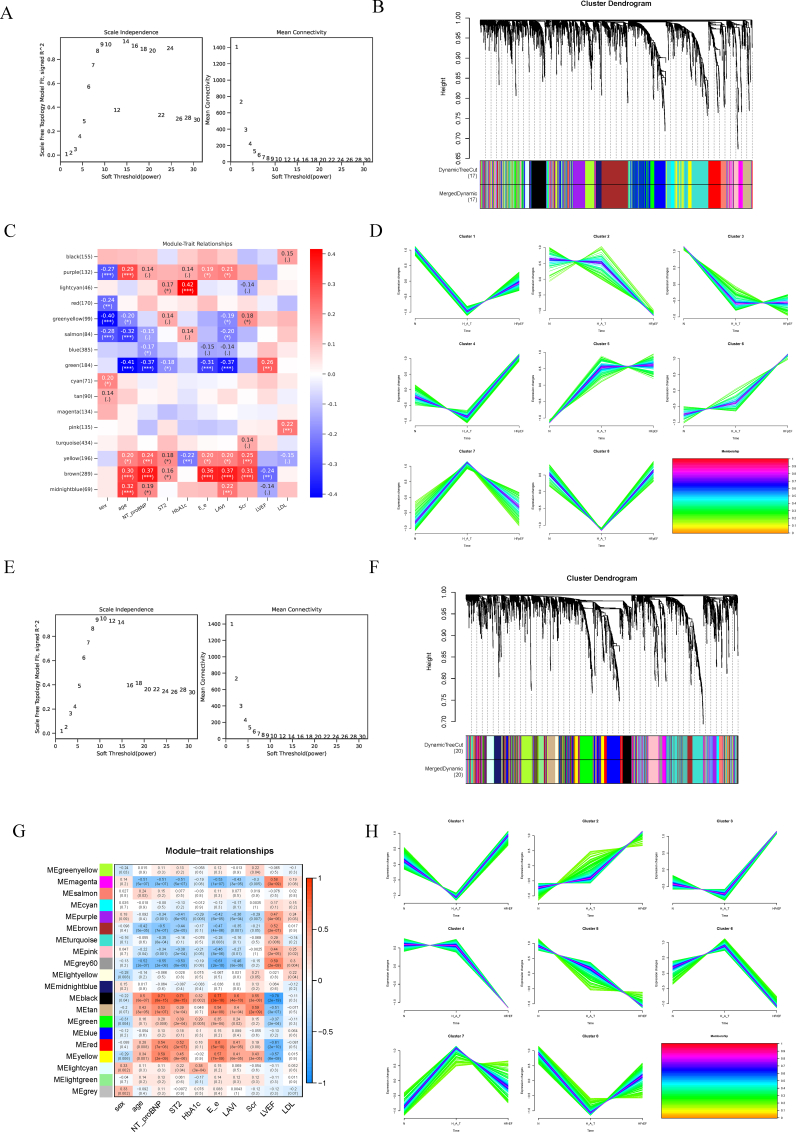
Weighted gene coexpression network analysis (WGCNA) in the N–H_A_T-HFpEF and N–H_A_T-HFrEF groups. A & E: Soft-threshold power selection for network construction in the N–H_A_T-HFpEF (A) and N–H_A_T-HFrEF (E) groups. A power of 7 was chosen on the basis of the scale-free topology model fit (left) and mean connectivity (right). B & F: Cluster dendrogram of metabolites showing distinct coexpression modules via dynamic tree cutting. C & G: Module‒trait relationship heatmaps. D & H: Trend analysis across the N, H_A_T, HFpEF and HFrEF groups.

To explore the potential relationships between gene coexpression modules and clinical traits, we conducted weighted gene coexpression network analysis (WGCNA) and trend analysis on data from the four groups. Using soft thresholding, we determined that a power value of 7 yielded a scale-free topology model fit of approximately 0.8, ensuring the network's scale-free topology and the rationality of module division (Fig. 5A and B).

In the N–H_A_T-HFpEF group, we applied the dynamic tree cut method to divide the metabolites into 17 coexpression modules, which were visualized through a cluster dendrogram (Fig. 5C). Each module represents a set of coexpressed metabolites. Through heatmap analysis, we demonstrated the correlations between these modules and clinical traits such as sex, age, NT-proBNP, ST2, HbA1c, and LVEF. Notably, the metabolites in the brown module were significantly positively correlated with NT-proBNP, E/e', and LAVI, whereas those in the green module were significantly negatively correlated with these clinical traits. These findings suggest that the brown and green modules may play critical regulatory roles in the pathophysiology of HFpEF.

In the N–H_A_T-HFrEF group, the metabolites were divided into 20 coexpression modules. Further analysis revealed that the metabolites in the black module were significantly positively correlated with NT-proBNP (r = 0.71, p < 0.001) and significantly negatively correlated with LVEF (r = −0.78, p < 0.001). These results suggest that the metabolites in the black module may play important roles in the regulation of cardiac function, particularly in the pathological progression of HFrEF (Fig. 5G).

To investigate the dynamic changes in metabolites across different conditions, we performed trend analysis on data from the N group, H_A_T group, and HFpEF group. The results revealed that the changes in the expression of metabolites across these three conditions could be categorized into eight distinct expression clusters, which highlights the potential roles of these metabolites in disease progression.

The metabolites in Cluster 2 exhibited a gradual increase in expression, progressing from the N group to the H_A_T group and then to the HFpEF group. These findings suggest that these metabolites may play a driving role in the transition from metabolic disorders to HFpEF, indicating their potential involvement in the pathophysiological mechanisms of heart failure (Fig. 5D).

In contrast, the metabolites in Cluster 6 presented increased activity under normal conditions (N group), but their expression progressively decreased in the H_A_T and HFpEF groups. This trend suggests that the activity of these metabolites may be suppressed during disease progression, suggesting that they play a key role in maintaining normal metabolic status and that their downregulation could be closely related to the pathogenesis of HFpEF.

Furthermore, in a separate dataset comprising the N group, H_A_T group, and HFrEF group, we observed that metabolites in Cluster 2 and Cluster 5 were closely associated with the development of HFrEF. The expression trends of these metabolites further support their critical roles in the progression of HFrEF (Fig. 5H).

### 3.6 Metabolite analysis and diagnostic efficacy in HFpEF, highlighting the heterogeneity of HFrEF

**Fig. 6:**
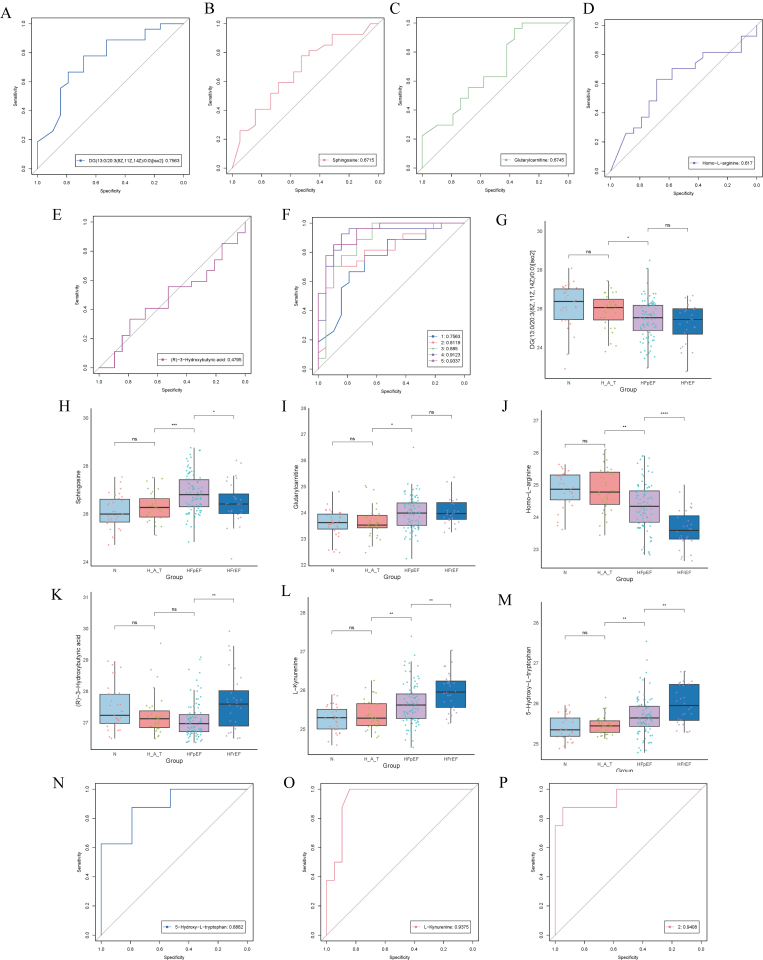
ROC curves and boxplots of key metabolites across groups. A-E & N–O: ROC curves showing the diagnostic performance of DG (13:0/20:3(8Z,11Z,14Z)/0:0) [iso2] (A), sphingosine (B), glutarylcarnitine (C), Homo-l-arginine (D), (R)-3-hydroxybutyric acid (E), 5-hydroxy-l-tryptophan (N), and l-kynurenine (O). F: Combined ROC analysis of A-E metabolites for enhancing HFpEF diagnosis. P: Combined ROC analysis of N and O. G–M: Boxplots comparing metabolite levels across groups: normal (N), hypertension with diabetes (H_A_T), HFpEF, and HFrEF. Statistical significance is indicated (∗p < 0.05, ∗∗p < 0.01, ns = nonsignificant).

Through multivariate analysis of metabolites across different groups (N, H_A_T, HFpEF, and HFrEF), we identified several significantly differentially abundant metabolites and evaluated their diagnostic efficacy in distinguishing heart failure subtypes via receiver operating characteristic (ROC) curves. The results revealed that DG (13:0/20:3(8Z,11Z,14Z)/0:0), glutarylcarnitine, homo-l-arginine, and sphingosine had AUC values of 0.798, 0.814, 0.871, and 0.814, respectively, in diagnosing HFpEF, whereas (R)-3-hydroxybutyric acid had a relatively lower AUC of 0.675, still demonstrating diagnostic value in specific pathological states. The combined analysis of these five metabolites further increased the AUC, highlighting the advantage of using multiple biomarkers to improve the diagnostic power for HFpEF. Box plot analysis revealed significant differences in metabolite expression levels across the groups, with l-kynurenine and 5-hydroxy-l-tryptophan showing notably higher levels in the HFrEF group than in the control group, indicating their potential specificity for HFrEF. These findings provide valuable insights into the metabolic heterogeneity of heart failure subtypes and underscore the importance of further investigations (Fig. 6).

## 4 Discussion

HFpEF and HFrEF exhibit significant differences in their underlying pathophysiology and epidemiological characteristics [9]. HFpEF is closely associated with metabolic syndrome, including conditions such as hypertension and diabetes, whereas HFrEF is more frequently linked to ischemic heart disease and myocardial injury [10]. Although previous studies have identified certain metabolic distinctions between the two, such as alterations in fatty acid oxidation, glucose metabolism, and branched-chain amino acid metabolism, comprehensive nontargeted metabolomic analyses of these heart failure subtypes remain limited. The relationships between these metabolic features and systemic metabolic states also require further clarification. Therefore, conducting nontargeted plasma metabolomic studies to explore the metabolic differences between HFpEF and HFrEF thoroughly could aid in identifying characteristic metabolites for HFpEF, thereby providing a more in-depth understanding of the distinct pathophysiological mechanisms of both conditions.

Clinical demographic studies have shown that HFpEF and HFrEF have distinct clinical characteristics, which are evident in aspects ranging from cardiac function and medication use to biomarker levels. First, patients with HFpEF and HFrEF are generally older, which is consistent with the increased risk of heart failure with increasing age. Although a slightly greater proportion of HFpEF patients were female, the sex difference did not reach statistical significance, which may be related to the protective effects of sex hormones. This observation aligns with the results of a cohort study involving 28,820 participants, where gender differences diminished after adjustment [10]. With respect to medication use, diuretics are notably prominent in the treatment of HFrEF patients to alleviate symptoms, reflecting their importance in reducing volume overload and managing symptoms.

Echocardiographic findings revealed differences in cardiac function between HFpEF patients and HFrEF patients. HFpEF patients exhibit diastolic dysfunction, as indicated by a preserved left ventricular ejection fraction (LVEF), but significantly elevated E/e' ratios and left atrial volume indices—characteristic markers of diastolic dysfunction. The H2FPEF score [11,12], a simple, noninvasive diagnostic tool for HFpEF, incorporates the E/e' ratio, effectively screening for HFpEF and providing a foundation for subsequent metabolomic investigations. In contrast, HFrEF patients show a marked reduction in LVEF, indicating more severe ventricular systolic dysfunction.

Laboratory data indicate that both HFpEF and HFrEF patients have significantly elevated NT-proBNP levels, with a more pronounced increase in HFrEF, reflecting greater cardiac stress and volume overload. Additionally, growth-stimulating express gene 2 (ST2), a prognostic biomarker of HFrEF, is significantly elevated in HFrEF patients, which may be associated with myocardial stress and fibrosis, highlighting its potential role in assessing the severity of heart failure [13].

Overall, these findings further support the differing clinical presentations and metabolic states of HFpEF and HFrEF patients. While HFpEF patients maintain near-normal ejection fractions, the presence of diastolic dysfunction and elevated cardiac stress markers suggest underlying myocardial pathophysiological changes. In contrast, HFrEF is characterized by a reduced ejection fraction and more pronounced myocardial stress.

In this study, we comprehensively integrated clinical information with metabolomic analysis, utilizing machine learning and random forest methods to identify key metabolites associated with HFrEF and HFpEF. Our findings demonstrate that metabolites related to inflammation, myocardial fibrosis, and oxidative stress are predominantly associated with HFrEF. In contrast, HFpEF is characterized primarily by disturbances in metabolic syndrome, energy metabolism, and systemic metabolic imbalance. Specifically, 5-hydroxytryptamine (5-HT) and l-kynurenine (KYN) were notably linked to HFrEF, whereas DG (13:0/20:3(8Z,11Z,14Z)/0:0), glutarylcarnitine, Homo-l-arginine, sphingosine/S1P, and (R)-3-hydroxybutyric acid (β-OHB) were strongly associated with HFpEF. These metabolites not only reflect the distinct pathophysiological mechanisms of the two heart failure subtypes but also highlight their different disease states, providing novel metabolic markers for differentiating HFrEF from HFpEF and offering potential avenues for targeted therapeutic strategies.

These metabolites are intricately involved in the pathophysiology of HFpEF, reflecting disturbances in metabolic regulation, energy metabolism, cellular signaling, and myocardial remodeling. DG(13:0/20:3(8Z,11Z,14Z)/0:0)[iso2], a type of diacylglycerol (DAG), acts as an intracellular second messenger and plays a pivotal role in cellular signaling pathways that govern growth, differentiation, and metabolic homeostasis [14]. In cardiac tissue, excess accumulation of DAG can induce mitochondrial and endoplasmic reticulum stress, activating downstream pathways such as protein kinase C (PKC) and mitogen-activated protein kinase (MAPK) [15]. This cascade promotes lipotoxicity, myocardial diastolic dysfunction, and fibrosis, key contributors to HFpEF progression.

Glutarylcarnitine serves as an intermediate in fatty acid oxidation and energy metabolism, closely linked to lysine and tryptophan catabolism [16]. Elevated plasma levels of glutarylcarnitine in metabolic disorders, such as glutaric acidaemia type I, indicate impaired fatty acid oxidation. potentially mirroring myocardial metabolic remodeling and bioenergetic deficiency in HFpEF.

Similarly, homo-l-arginine, significantly reduced in HFpEF patients [17], is integral to nitric oxide (NO) synthesis. Its decline may reduce NO bioavailability, exacerbate endothelial dysfunction, and increase myocardial stiffness—hallmarks of HFpEF pathophysiology.

Sphingosine and its phosphorylated derivative, sphingosine-1-phosphate (S1P), are central to cardiovascular regulation. S1P mediates cardioprotective effects via PI3K/Akt and ERK1/2 pathways, safeguarding against myocardial ischemia-reperfusion injury, modulating fibrosis, and preserving endothelial integrity [[18], [19], [20]]. Given the chronic low-grade inflammation and vascular abnormalities in HFpEF, alterations in S1P signaling likely play a pathophysiological role beyond mere associations with blood pressure or BMI [21].

(R)-3-Hydroxybutyric acid (β-OHB), a ketone body produced during fatty acid oxidation, becomes a compensatory fuel under metabolic stress conditions such as fasting, diabetes, or heart failure [22]. In HFpEF, β-OHB serves as an alternative energy source for cardiomyocytes, helping to improve myocardial energy metabolism and support mitochondrial function, thus reducing oxidative stress, offering an endogenous mechanism to preserve myocardial energy balance [23]. Pharmacological interventions, such as SGLT2 inhibitors (e.g., dapagliflozin) [24].

Taken together, these metabolic alterations highlight profound shifts in substrate utilization, cellular signaling, and structural remodeling that underpin HFpEF. While they hold promise as potential biomarkers to complement traditional markers like NT-proBNP [25]-potentially improving diagnostic specificity in distinguishing HFpEF from HFrEF, especially in metabolic syndrome-this study primarily underscores their roles in disease mechanisms. Further research is warranted to explore the specific mechanisms of these metabolites in HFpEF, validating their clinical utility and providing new insights into the diagnosis and treatment of HFpEF.

HFrEF and HFpEF exhibit distinct metabolic and pathological characteristics. HFrEF is primarily associated with metabolic imbalances linked to inflammation and myocardial fibrosis. This distinction is particularly evident in metabolites such as 5-HT and KYN. Relationship between 5-HT and HFrEF: 5-HT, the direct metabolite of 5-hydroxy-l-tryptophan (5-HTP), plays a crucial role in HFrEF [26]. In HFrEF patients, metabolic abnormalities in 5-HT are closely linked to myocardial fibrosis and vascular dysfunction [27]. By interacting with the 5-HT2B receptor, 5-HT promotes myocardial fibrosis [28], leading to increased myocardial stiffness and pathological remodeling, thereby exacerbating cardiac dysfunction. Moreover, 5-HT can induce vasoconstriction and increase peripheral vascular resistance, further increasing the cardiac load [29]. These characteristics reflect the extensive inflammatory state in HFrEF and its impact on myocardial and vascular structure, which contrasts with the metabolic abnormalities observed in HFpEF.

Relationship between KYN and HFrEF: KYN is a key intermediate in tryptophan metabolism and plays a role in HFrEF related to inflammation and myocardial fibrosis [30]. During inflammatory responses, cytokines can induce IDO activity, increasing KYN production. KYN affects calcium homeostasis and mitochondrial function in cardiomyocytes, leading to oxidative stress and causing damage to cardiomyocytes and endothelial cells, thereby promoting myocardial fibrosis and cardiac remodeling [31]. Additionally, KYN modulates vascular tone and hemodynamics, impacting vascular function. Its metabolic imbalance is associated with inadequate energy supply in cardiomyocytes, a phenomenon particularly evident in HFrEF patients [32,33]. Elevated KYN levels indicate a state of chronic immune activation and inflammation and are correlated with the severity and prognosis of heart failure, thus serving as a potential biomarker [34].

In HFrEF, the dysregulation of 5-HT and KYN metabolism is associated mainly with inflammation, oxidative stress, and myocardial fibrosis, which exacerbate the pathological remodeling of myocardial and vascular structures, highlighting an inflammation-centric pathophysiology. In contrast, the metabolic profile of HFpEF patients is related primarily to metabolic syndrome and disturbances in myocardial energy metabolism, which are characterized by reduced metabolic efficiency and systemic metabolic imbalances. Metabolites such as DAG, glutarylcarnitine, homo-l-arginine, sphingosine/S1P, and β-OHB reflect these metabolic abnormalities in HF.

The key metabolites identified in this study hold significant potential not only for diagnosing HFpEF but also for providing insights into novel personalized treatment strategies through further exploration of their metabolic pathways. In particular, for patients with diabetes and hypertension, these metabolites could serve as early indicators of heart failure progression, supporting individualized management and intervention. However, this study has several limitations. First, the relatively small sample size and single-center design may limit the generalizability of the findings. Therefore, larger multicenter studies are needed to validate the diagnostic efficacy of these biomarkers further. Second, the cross-sectional design precludes the assessment of dynamic changes in metabolites throughout disease progression. Longitudinal studies will be necessary to clarify the role of these metabolites in the development of heart failure. Finally, although key biomarkers have been identified, their specific biological mechanisms remain to be experimentally validated, which is essential for determining their potential as therapeutic targets. Additionally, we did not systematically collect detailed dietary or physical activity data, which are known to significantly influence metabolites such as β-hydroxybutyrate and acylcarnitines; this omission limits our ability to fully disentangle lifestyle-related metabolic adaptations from disease-specific alterations. Moreover, while we carefully recorded medication use, including loop diuretics in the majority of HFrEF patients, we did not adjust for medication effects, nor did we incorporate eGFR adjustments despite screening to exclude overt renal dysfunction. Similarly, although all samples were collected under rigorously standardized protocols-following an overnight fast, immediate ice processing, snap-freezing, and identical batch thawing-the time from diagnosis to sampling was not stratified, which could also introduce residual variability.

Future research should focus on validating the diagnostic efficacy of these metabolites in larger, multicenter cohorts while also assessing their specificity and applicability across diverse populations. Integrating multivariable adjustments for medications and renal function, along with standardized dietary assessments and objective activity monitoring, will be critical to refine these associations. Moreover, integrating standardized dietary assessments and objective activity monitoring, alongside mechanistic experimental studies, will be crucial to elucidate the underlying biological pathways and establish their potential role in personalized therapy.

## 5 Conclusions

This study, which used metabolomics combined with machine learning, identified several key metabolites associated with HFpEF, showing the potential to enhance diagnostic specificity. These metabolites, when combined with traditional markers such as NT-proBNP, offer a more comprehensive perspective for the early diagnosis of HFpEF. These findings provide important insights for further exploration of the metabolic mechanisms underlying HFpEF and its potential for personalized treatment strategies.

## CRediT authorship contribution statement

**Qipeng Jin:** Conceived and designed the study, analyzed the data and wrote the paper. **Zhicheng Wang:** Performed the research and contributed new methods or models. **Wenyong Lin:** Performed the research and analyzed the data. **Chunling Zhang:** Contributed new methods or models and analyzed the data. **Xiaolong Wang:** Conceived or designed the study, contributed new methods or models and wrote the paper.

## Notes

This study protocol was approved by the Ethics Committee of Shuguang Hospital, Affiliated with the Shanghai University of Traditional Chinese Medicine (approval number: 2023-1345-112, Date of Approval: August 2, 2023). All participants voluntarily agreed to participate after fully understanding the study's content and objectives and consented to the publication of their data and/or images in this article. Trial Registration: China Clinical Trial Registration Center, ChiCTR2400082425. Registered on March 28, 2024.

## Declaration of competing interest

The authors declare that they have no known competing financial interests or personal relationships that could have appeared to influence the work reported in this paper.

## Data Availability

Data will be made available on request.

## Abbreviations

ACEi: Angiotensin-converting enzyme inhibitor
ARB: Angiotensin II receptor blocker
CCB: Calcium channel blocker
E/e' ratio: Ratio of early mitral inflow velocity to early mitral annular velocity
HFpEF: Heart failure with preserved ejection fraction
HFrEF: Heart failure with reduced ejection fraction
LAVI: Left atrial volume index
LVEF: Left ventricular ejection fraction
NT-proBNP: N-terminal pro b-type natriuretic peptide
NOACs: New Oral Anticoagulants
ST2: Growth stimulating express gene 2
WGCNA: Weighted gene coexpression network analysis
ROC: Receiver operating characteristic
S1P: Sphingosine-1-phosphate
