# Supplementary material for: Exploration of serum biomarkers in heart failure patients with preserved and reduced ejection fractions through analysis of heterogeneity
Source: Biochem Biophys Rep. 2025 Jul 31;43:102183. doi: 10.1016/j.bbrep.2025.102183 (PMC12335954; doi:10.1016/j.bbrep.2025.102183)
Supplement: Multimedia component 2 [file mmc2.docx]

### ****Supplementary Information****

#### ****HFpEF diagnostic criteria following the 2021 ESC Guidelines****

1. Presence of typical symptoms and/or signs of heart failure.
2. Left ventricular ejection fraction (LVEF) is normal or slightly reduced (LVEF ≥50%).
3. Brain natriuretic peptide (BNP) > 80 pg/mL or NT-proBNP > 220 pg/mL.
4. Recent echocardiogram and/or cardiac MRI before enrollment showing evidence of LVEF > 50% and structural heart disease (such as left ventricular hypertrophy or left atrium (LA) enlargement). Structural heart disease is defined as:
   - LA width (diameter) ≥ 3.8 cm or
   - LA length ≥ 5.0 cm or
   - LA area ≥ 20 cm² or
   - LA volume ≥ 55 mL or
   - LA volume index ≥ 29 mL/m².

#### ****Inclusion Criteria****

1. Diagnosed with heart failure with preserved ejection fraction (HFpEF) according to the above criteria.
2. Age between 40 and 80 years.
3. Complete patient data, capable of adhering to medication, and able to comply with follow-up requirements.
4. The patient and their family have been informed of the study’s purpose, signed the informed consent, and voluntarily agreed to participate.

#### ****Exclusion Criteria****

1. Patients with restrictive (infiltrative) cardiomyopathy or congenital heart disease.
2. Acute coronary syndrome.
3. History of stent placement, coronary artery bypass grafting, or permanent pacemaker implantation within 90 days prior to enrollment (from the time of hospital admission).
4. Estimated glomerular filtration rate (eGFR) ≤ 20 ml/min/1.73 m² or severe liver dysfunction.
5. Patients with malignant tumors, hematologic diseases, or acute infectious diseases.
6. Patients with psychiatric disorders or infectious diseases.

#### ****Exclusion After Enrollment Criteria****

1. If the patient selection violates the inclusion or exclusion criteria discovered after trial initiation.
2. Non-compliance, defined as medication adherence <80% or >120%.
3. The patient took prohibited medications as outlined in the study protocol during the observation period.

#### ****Withdrawal Criteria****

1. Withdrawal determined by the investigator.
2. Worsening of the patient’s condition during the trial, which may lead to dangerous events. The investigator may decide to terminate the clinical trial for the safety of the patient, and the patient may receive other treatments, with the case considered as ineffective.
3. Occurrence of complications, comorbidities, or significant physiological changes making it unsuitable to continue the trial.
4. Occurrence of adverse events or serious adverse events.
5. The patient voluntarily decides to withdraw from the trial.
6. The patient withdraws from the clinical trial without providing any reason.
7. The patient does not explicitly withdraw but stops taking the medication or attending follow-up, leading to a loss of contact.

In all cases, every effort should be made to understand and document the reasons for withdrawal. If the patient feels the treatment is ineffective, experiences unbearable side effects, or has personal issues preventing continuation, this should be noted.
